# Supercapacitor Materials: Structure, Properties, and Applications for Energy Storage in Engineering Systems

**DOI:** 10.3390/ma19122454

**Published:** 2026-06-08

**Authors:** Lincoln Pinoski, Subin Antony Jose, Jacob Dowling, Nicholas Eastwood, Carly Farthing, Gavin Fisher, Pradeep L. Menezes

**Affiliations:** Department of Mechanical Engineering, University of Nevada, Reno, NV 89557, USA; lpinoski@unr.edu (L.P.); subinj@unr.edu (S.A.J.); jacobdowling@unr.edu (J.D.); neastwood@unr.edu (N.E.); cfarthing@unr.edu (C.F.); gavinfisherprofessional@gmail.com (G.F.)

**Keywords:** supercapacitors, energy storage systems, electrode materials, pseudocapacitance, hybrid supercapacitors

## Abstract

The increasing global demand for high-performance, reliable, and sustainable energy storage systems has accelerated the development of supercapacitors as technologies capable of bridging the performance gap between conventional capacitors and batteries. Supercapacitors combine rapid charge–discharge capability, high power density, and exceptional cycle life through charge storage mechanisms based on ion adsorption and fast surface redox reactions at the electrode–electrolyte interface. This review examines the fundamental operating principles, charge storage mechanisms, electrode materials, mechanical and functional properties, fabrication methods, and engineering applications of modern supercapacitors. Carbon-based materials, metal oxides, conducting polymers, MXenes, sulfides, nitrides, borides, and emerging hybrid systems are critically compared in terms of capacitance, energy density, cycling stability, and mechanical robustness. Additionally, recent advances in scalable manufacturing approaches, including thin-film deposition and printing technologies, are discussed alongside key challenges such as limited energy density, interfacial instability, mechanical degradation, electrolyte compatibility, and large-scale processing. By consolidating recent developments across materials science, electrochemistry, and device engineering, this review provides insight into future directions for next-generation high-performance supercapacitor technologies.

## 1. Introduction

The global demand for efficient and sustainable energy storage technologies is growing rapidly as renewable energy systems, electric vehicles, portable devices, and advanced electronics continue to develop. Traditional energy storage devices, such as batteries and capacitors, each have inherent limitations. Batteries offer high energy density but relatively slow charge–discharge rates, while conventional capacitors deliver rapid power but store relatively little energy [1]. Engineers and scientists are tasked with bridging the gap between these predominant energy storage methods and developing new technologies to power the ongoing technological revolution. The effectiveness of any battery-powered device is directly related to how fast, efficient, and sustainable its power supply is. Traditional batteries store energy chemically, allowing high capacity, but they are slow and heavy, limiting their use in many applications. Traditional capacitors store energy electrostatically, making them fast, but with very low energy storage ability [1]. Supercapacitors bridge this gap by combining desirable features of both systems. They provide exceptional power density, rapid charge/discharge capability, long cycle life, and excellent efficiency, all of which make them valuable for modern engineering applications [1]. As industries seek lightweight, durable, and scalable solutions for energy storage, supercapacitors are becoming increasingly relevant in fields ranging from electric vehicles and robotics to wearable technology and renewable energy systems [2]. Their versatility and performance advantages make them a key focus of materials science and mechanical engineering research, particularly as new materials and fabrication methods continue to improve performance. Indeed, supercapacitors have great potential in mechanical engineering and materials science as lightweight energy storage mechanisms with fast charging capabilities. Current and emerging applications include electric and hybrid vehicles, wearable devices, and robotics [2]. Table 1 contains a detailed comparison between batteries and supercapacitors.

The world of supercapacitor innovation is opening new avenues for technological progress in a range of sectors, from the smallest portable devices to the most complex systems. This work covers the basic working principles of supercapacitors, the materials they employ, and the mechanical and functional properties that make them so intriguing. Different forms of supercapacitors, including carbon-based, metal oxide, conducting polymer, and hybrid systems, are compared. The unique properties that distinguish supercapacitors are examined to elucidate how this technology will be an asset in the future. Additionally, we review their fabrication processes, current applications, future challenges that must be overcome, and avenues for further adoption.

## 2. Principles of Supercapacitors

Supercapacitors are energy storage devices that operate on principles fundamentally different from both traditional dielectric capacitors and chemical batteries. Unlike batteries, which store energy through slow chemical reactions occurring throughout the bulk of electrode materials, supercapacitors store energy through processes occurring primarily at the surface of the electrodes. This surface-based mechanism allows supercapacitors to achieve extremely fast charge and discharge cycles and cycle lives ranging from hundreds of thousands to over one million cycles [6]. Because they do not depend on diffusion-limited chemical phase transformations, supercapacitors can respond in milliseconds, making them ideal for applications requiring rapid bursts of energy. These attributes are why supercapacitors are increasingly viewed as a promising energy storage solution for next-generation technologies.

### 2.1. Working Mechanism

**Figure 1:**
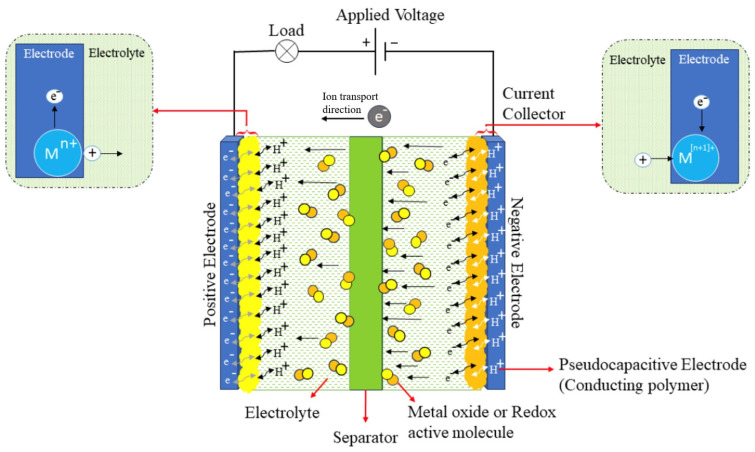
Schematic of ion movement and charge storage in a pseudocapacitor. Reproduced from [4], World Electr. Veh. J. MDPI 2024.

At the fundamental level, a supercapacitor operates via electrostatic or faradaic charge storage at the electrode–electrolyte interface. When a voltage is applied, ions in the electrolyte migrate toward the oppositely charged electrode surfaces, forming a charge-separation layer known as the electrical double layer [7]. This phenomenon is analogous to a conventional capacitor but occurs at the nanoscale within the pores of the electrode material, dramatically increasing the available surface area for charge storage [4]. Because the primary charge storage involves physical ion adsorption rather than bulk chemical reactions, the energy storage is highly reversible and efficient. This leads to extremely long operational lifetimes and minimal degradation compared to chemical batteries. Additionally, the absence of large-scale structural changes in the electrode materials allows supercapacitors to charge and discharge rapidly, with minimal heat generation and high round-trip efficiency [8]. Figure 1 illustrates the working principle of a pseudocapacitor, a type of supercapacitor. An electrode on the right side made of a conducting polymer transfers ions through the electrolyte and electrolyte separator to the positive electrode, allowing current to flow to the load.

The charge storage kinetics differ significantly among electric double-layer capacitors (EDLCs), pseudocapacitors, and intercalation-type hybrid capacitors. In EDLCs, charge storage occurs through rapid electrostatic adsorption of ions at the electrode–electrolyte interface without charge transfer reactions, resulting in exceptionally fast ion transport kinetics and high power density [9]. Because no bulk structural changes occur within the electrode material, EDLCs exhibit excellent cycling stability and near-instantaneous charge–discharge behavior. In contrast, pseudocapacitors store charge through fast and reversible faradaic redox reactions occurring at or near the electrode surface. Although these reactions introduce charge-transfer resistance and somewhat slower kinetics compared to EDLCs, pseudocapacitors achieve significantly higher capacitance and energy density due to electron-transfer-based storage mechanisms [10]. Intercalation-type hybrid capacitors involve ion diffusion into the bulk crystal lattice of the electrode material, similar to battery-type behavior. As a result, their charge storage kinetics are primarily diffusion-controlled rather than surface-controlled, leading to slower charge–discharge rates but substantially higher energy storage capability. Consequently, EDLCs generally provide the highest power density and cycling stability, pseudocapacitors offer a balance between power and energy density, and intercalation-type hybrid capacitors bridge the performance gap between conventional supercapacitors and rechargeable batteries [10].

### 2.2. Types of Supercapacitors

Supercapacitors are broadly categorized into two types based on their charge storage mechanism: EDLCs and pseudocapacitors. EDLCs rely on the formation of the electrical double layer at the interface between a high-surface-area electrode and the electrolyte. Materials such as activated carbon, graphene, carbon nanotubes, metal matrix composites, and MXenes are commonly used due to their large surface area, high conductivity, and chemical stability [11]. EDLCs excel in power density and cycle life but typically have lower energy density compared to batteries. Pseudocapacitors, on the other hand, store energy through fast and reversible faradaic (redox) reactions occurring at or near the electrode surface. Common pseudocapacitive materials include transition metal oxides like MnO_2_ and RuO_2_, as well as conducting polymers such as polyaniline (PANI) and polypyrrole (PPy) [12]. These materials enable higher energy densities than EDLCs but often suffer from lower cycle life due to structural changes during repeated redox cycling.

**Figure 2:**
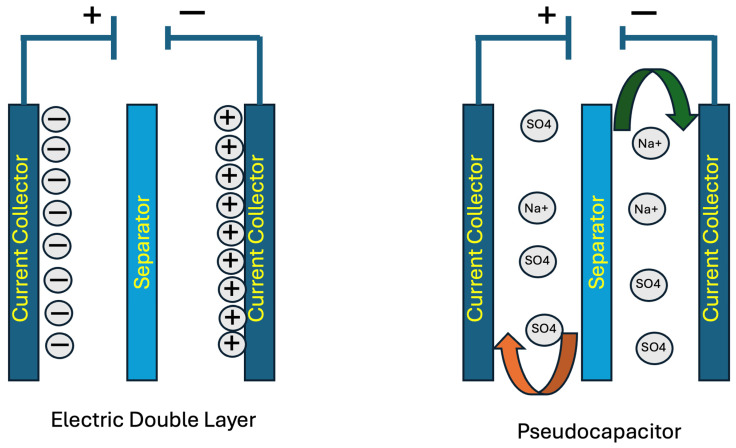
Working principle and differences between pseudocapacitor and EDLC. Arrows represent the circulation of electrolyte ions during reactions.

Researchers are also exploring hybrid supercapacitors that integrate features of both EDLCs and pseudocapacitors. By combining the fast charge/discharge capability and durability of EDLCs with the higher energy storage of pseudocapacitive electrodes, hybrid systems aim to deliver balanced performance suitable for a wider range of applications [13]. For example, an asymmetric device may use an activated carbon electrode paired with a faradaic electrode to significantly boost energy density while maintaining high power output. Figure 2 illustrates the fundamental charge storage mechanisms of EDLCs and pseudocapacitors. In EDLC systems, energy is stored through electrostatic ion adsorption at the electrode–electrolyte interface, represented as the particles on the current collectors, whereas pseudocapacitors additionally involve fast and reversible faradaic redox reactions with electrolyte ions like Na^+^ and SO_4_, enabling higher capacitance and energy storage capability.

### 2.3. Key Performance Metrics

Evaluating supercapacitor performance involves several key metrics that reflect their capabilities and limitations. Energy density (Wh/kg) is the total energy stored per unit mass. Supercapacitors typically have lower energy density than batteries, but advances in pseudocapacitive materials and hybrid designs have improved this metric in recent years [14]. Power density (W/kg) indicates the rate at which energy can be delivered; supercapacitors far outperform batteries in power density, often delivering 10–100 times greater power [6]. Cycle life, the number of charge/discharge cycles a device can undergo before significant performance degradation, is another crucial factor. Supercapacitors can often exceed 1,000,000 cycles with minimal capacity loss [15]. Response time refers to how quickly the device can deliver its stored energy. Since charge storage in supercapacitors is primarily surface-based, they can respond in milliseconds. Efficiency (often measured as coulombic efficiency) is typically very high (>90%) due to minimal resistive losses and the lack of slow chemical reactions. Finally, mechanical stability is increasingly important for emerging applications like flexible and wearable devices. Electrode materials such as MXenes and nanostructured cellulose composites offer enhanced flexibility and durability, enabling bending or stretching without loss of performance [16]. In addition to capacitance and energy density, practical supercapacitor performance is strongly influenced by self-discharge behavior, impedance characteristics, and long-term degradation mechanisms [17]. Self-discharge can occur through charge redistribution, parasitic faradaic reactions, or internal leakage pathways, reducing stored energy over time, while impedance behavior governed by ion diffusion resistance and charge-transfer resistance directly affects power delivery and rate capability [18]. Furthermore, degradation mechanisms such as electrode cracking, pore collapse, electrolyte decomposition, and interfacial instability can progressively increase internal resistance and reduce capacitance during extended cycling, making these factors critical considerations in engineering design and reliability analysis.

Supercapacitor performance is significantly influenced by thermal effects and structural integrity. Rapid charge/discharge cycles can generate heat, so materials with high thermal conductivity and stable pore structures are favored to dissipate heat and maintain performance. Likewise, the electrode structure must remain robust under repeated cycling. Mechanical failures like cracking, delamination, or pore collapse will reduce lifespan and capacitance [19]. Mechanical engineering considerations come into play when integrating supercapacitors into systems that undergo stress, vibration, or deformation. For instance, flexible supercapacitors for wearable devices must maintain electrical performance under bending or stretching. Advances in composite electrode structures and novel device architectures are helping meet these mechanical demands.

Recent research in supercapacitor technology focuses on improving energy density without sacrificing power density or cycle life. This includes the development of novel two-dimensional materials such as MXenes, which offer a combination of high conductivity, tunable surface chemistry, and mechanical strength [16]. Hybrid and asymmetric supercapacitors, which pair different electrode materials or combine battery-like and capacitor-like behavior, are gaining traction for applications requiring both high energy and high power [13,20]. Additionally, new fabrication techniques such as atomic layer deposition, 3D printing, and roll-to-roll processing are enabling more scalable and cost-effective production of supercapacitors [19]. These approaches are critical for integrating supercapacitors into commercial products ranging from electric vehicles and regenerative braking systems to industrial energy storage modules.

The electrolyte is an important factor that governs ion mobility and the cell’s voltage window. Electrolytes are commonly aqueous, organic, or ionic liquids, each offering trade-offs in conductivity, voltage range, and thermal stability. Aqueous electrolytes provide high ionic conductivity and low internal resistance but are limited to about 1 V per cell due to water’s decomposition voltage. Organic electrolytes extend the operational voltage to around 2.7–3.0 V at the cost of reduced conductivity and increased flammability. Recently, ionic liquid electrolytes have gained attention for their wide electrochemical stability window and non-volatile nature, enabling improved energy density and safer high-temperature operation [7,21].

The electrochemical performance of supercapacitors is strongly influenced by the interactions occurring at the electrode–electrolyte interface [22]. Electrolyte selection affects ion transport kinetics, voltage window, interfacial resistance, capacitance, and long-term cycling stability. In EDLCs, electrolyte ion size and solvation behavior determine how effectively ions can access porous carbon structures, directly influencing charge storage efficiency [9,23]. Aqueous electrolytes such as KOH and H_2_SO_4_ provide high ionic conductivity and rapid charge transport but are limited by narrow voltage windows due to water decomposition. In contrast, organic electrolytes and ionic liquids enable significantly higher operating voltages and energy densities, though their higher viscosity and lower conductivity can reduce rate capability [23,24]. Electrolyte chemistry is particularly critical for pseudocapacitive materials such as MnO_2_, RuO_2_, conducting polymers, and MXenes, where electrolyte ions actively participate in reversible redox reactions. Improper electrolyte-electrode compatibility can accelerate degradation mechanisms, including oxide dissolution, polymer swelling, surface oxidation, gas evolution, and interfacial instability. Consequently, optimization of supercapacitor performance increasingly relies on integrated electrode–electrolyte engineering approaches that simultaneously consider material structure, ion transport behavior, electrochemical stability, and device operating conditions [25].

Further improvements in energy density are being realized with asymmetric/hybrid supercapacitors, which pair a capacitor-type electrode, like activated carbon, with a battery-type electrode such as lithium-intercalation material or metal oxide. This configuration leverages the fast response of double-layer charge storage on one side and the higher capacity faradaic reactions on the other. Such hybrid systems have demonstrated two to three times the energy density of pure EDLCs while preserving high cycle life [20,26]. Additionally, flexible and micro-scale supercapacitor designs are emerging for wearable and biomedical devices, where mechanical deformability and miniaturization are critical [27].

To summarize how supercapacitors compare to other energy storage systems, Table 2 highlights their operational differences relative to batteries, particularly in cycle life, energy density, power density, and charging behavior. These distinctions are essential when selecting suitable energy storage for various engineering applications.

### 2.4. Electrochemical Characterization

Electrochemical characterization techniques are essential for evaluating the charge storage behavior, rate capability, impedance response, and cycling stability of supercapacitors. Commonly employed methods include cyclic voltammetry (CV), galvanostatic charge–discharge (GCD), and electrochemical impedance spectroscopy (EIS), each providing insight into different aspects of electrochemical performance [29,30,31]. These techniques are widely used to distinguish electric double-layer capacitance from pseudocapacitive and diffusion-controlled charge storage mechanisms.

Cyclic voltammetry is one of the most frequently used techniques for analyzing supercapacitor behavior because it provides rapid insight into charge storage mechanisms and reversibility. Ideal EDLC materials typically exhibit nearly rectangular CV curves due to rapid electrostatic ion adsorption, whereas pseudocapacitive materials display redox peaks associated with faradaic reactions. The shape distortion of CV curves at high scan rates can also indicate ion transport limitations and internal resistance effects. Additionally, analysis of current response as a function of scan rate enables separation of capacitive and diffusion-controlled contributions to charge storage behavior [32].

Galvanostatic charge–discharge measurements are commonly used to evaluate specific capacitance, energy density, power density, coulombic efficiency, and cycling stability. Ideal EDLC systems generally produce nearly triangular charge–discharge curves with minimal voltage drop, while pseudocapacitive materials may exhibit nonlinear profiles due to redox reactions [33]. The internal resistance of the device is often estimated from the instantaneous IR drop observed at the beginning of discharge. Long-term cycling studies using repeated GCD testing are also critical for evaluating degradation behavior and electrode stability.

Electrochemical impedance spectroscopy provides detailed information regarding charge-transfer resistance, ion diffusion behavior, electrolyte resistance, and interfacial processes across a wide frequency range [34]. Nyquist plots are commonly used to analyze equivalent series resistance, semicircular charge-transfer regions, and low-frequency diffusion-controlled behavior. EIS is particularly valuable for studying porous electrode architectures and electrolyte-electrode interactions because it reveals how ion transport and conductivity change during cycling or under different operating conditions.

## 3. Materials for Supercapacitors

The performance of a supercapacitor is largely determined by the materials used for its electrodes. An ideal electrode material provides a high surface area for charge storage, good electrical conductivity, stability over many charge–discharge cycles, and, in many cases, additional faradaic activity. Supercapacitor electrode materials can be broadly grouped into carbon-based materials, metal oxides, conducting polymers, and composite-hybrid materials, each offering distinct advantages and challenges. In this section, we review these material classes, their properties, and recent advances in their development.

### 3.1. Carbon-Based Materials

Carbon-based materials have been the foundational electrode materials for commercial supercapacitors (EDLCs) for several decades. Activated carbon (AC), graphene, and carbon nanotubes (CNTs) are among the most pivotal examples in this category. Activated carbon, with its extremely high specific surface area (typically 1000–3000 m^2^/g), is the most widely used supercapacitor electrode material due to its abundance, low cost, chemical inertness, and excellent electrical conductivity [11]. A typical activated carbon electrode yields a specific capacitance on the order of 100 F/g in an aqueous electrolyte [35]. Despite its advantages, activated carbon is limited by relatively low intrinsic pseudocapacitance, which constrains the achievable energy density. Researchers have explored novel sources of activated carbon to improve performance. For instance, biomass-derived carbons from banana and tobacco stems are chosen for their high nitrogen and lignin content. These sources have achieved specific capacitances around 180 F/g, lower than some synthetic materials, but very economical as they derive from waste products [19,20]. Proper activation by using ZnCl_2_ vs. KOH can significantly influence pore structure and performance [36].

Graphene, a single-atom-thick sheet of carbon, offers exceptional electrical conductivity, mechanical strength, and a high theoretical surface area of 2630 m^2^/g [37]. Graphene-based electrodes can achieve higher capacitance than conventional activated carbon by offering a more accessible surface for ion adsorption. A key challenge, however, is preventing graphene sheets from restacking, which reduces accessible surface area and conductivity. Methods to address this include creating defects-pores in graphene sheets or assembling them into airy structures like foams or aerogels. Notably, composites like nickel cobaltite or carbon aerogels have leveraged graphene’s properties to reach capacitances of 1700 F/g in laboratory studies [38]. Advanced activated carbons and graphene materials have demonstrated energy densities up to 82 Wh/kg in organic electrolytes with corresponding power densities still in the kW/kg range, by engineering pore structures and surface chemistry, though performance can drop when electrode mass loading is increased [39]. Carbon nanotubes (CNTs) are another carbon allotrope investigated for supercapacitors. CNTs provide a conductive network and cylindrical pores, but their tendency to bundle via van der Waals forces and the difficulty of controlling their pore size distribution have posed challenges. Engineering CNT electrodes requires careful control of tube alignment, spacing, and integration with other materials to maximize their surface area and minimize resistance [40].

Beyond these classical carbons, researchers have developed templated porous carbons and carbons derived from metal–organic frameworks (MOFs) to push the limits of double-layer capacitance. MOF-derived carbons can have ultra-high surface areas and tuned pore architectures. For example, a nitrogen-enriched porous carbon derived from a MOF achieved an extraordinary specific capacitance of 2727 F/g at 1 A/g current density, and a composite of MOF-derived carbon with MnO_2_ showed 94% capacitance retention after 5000 cycles along with an energy density of 15.5 Wh/kg [41]. However, the complex and time-consuming synthesis of such materials remains a barrier to large-scale industrial use.

An emerging strategy to enhance carbon electrodes is to introduce a small degree of pseudocapacitance via surface functionalization or heteroatom doping. By doping carbon structures with heteroatoms such as nitrogen, boron, oxygen, and phosphorus, researchers have introduced faradaic reactions that can contribute as much as 40% of the total capacitance, effectively marrying some battery-like behavior with the electric double layer storage [39]. The challenge is to do this without sacrificing the long cycle life and power performance of pure carbon.

MXenes are a relatively new class of two-dimensional carbides and nitrides, which have garnered significant attention as supercapacitor materials. MXenes offer high metallic electrical conductivity, around 9880 S/cm for Ti_3_C_2_, and intrinsic pseudo-capacitance due to their redox-active surface terminations [42]. Pure MXene films can achieve volumetric capacitances on the order of 300 F/cm^3^ but tend to suffer from restacking and ion transport limitations at high rates, leading to performance degradation. Hybridizing MXenes with carbon nanostructures has proven highly effective in mitigating these issues. For example, Ti_3_C_2_ MXene integrated with CNTs in a layered film achieved 300 F/g at 1 A/g with 92% capacitance retention after 10,000 cycles at 20 A/g [43]. Similarly, MXene-graphene hybrid hydrogels have demonstrated gravimetric capacitances of 654 F/g with <2% decay over 8000 cycles, and MXene-based asymmetric devices have reached energy densities around 50 Wh/L with power densities of 127 kW/L [43]. These results highlight MXene-carbon hybrids as a promising path toward next-generation electrodes. However, challenges such as the chemical stability and the scalability of their synthesis, which often involves hazardous etchants like HF, must be addressed [43].

In summary, carbon-based materials are indispensable in supercapacitors for providing high power and long life, especially activated carbons and their advanced derivatives. Ongoing innovations ranging from biomass-derived carbons to graphene architectures and MXene hybrids aim to overcome carbon’s limitations in energy density while preserving its strengths. The electrochemical performance values are summarized in Table 3, which represent typical literature-reported ranges obtained under varying testing conditions. Reported capacitance, energy density, and power density values are strongly influenced by electrolyte composition, voltage window, current density, mass loading, and cell configuration. Therefore, direct quantitative comparison between studies should be interpreted cautiously.

An important factor in supercapacitor evaluation is the choice between three-electrode and two-electrode cell configurations. Three-electrode systems are widely used for fundamental material evaluation because they isolate the behavior of a single working electrode relative to a reference electrode. However, three-electrode measurements often report significantly higher capacitance values than practical full-cell devices because they do not fully account for device-level resistance, electrode balancing, or operational limitations [45]. In contrast, two-electrode configurations more accurately represent real supercapacitor performance and are generally preferred for evaluating practical energy storage capability. Electrode mass loading also strongly affects electrochemical performance [46]. Many laboratory-scale studies utilize ultralow active material loadings to maximize ion accessibility and minimize diffusion limitations, often resulting in exceptionally high reported capacitances. However, as mass loading increases toward commercially relevant values, ion transport resistance, electrode thickness effects, and incomplete electrolyte penetration can reduce accessible surface area and lower capacitance retention [47]. Therefore, performance values obtained at extremely low mass loadings may not accurately represent practical device behavior. Current density and scan rate similarly influence reported electrochemical metrics. At low current densities or slow scan rates, electrolyte ions have sufficient time to access internal pores and redox-active sites, typically producing higher measured capacitance values. At higher charging rates, ion diffusion limitations and increased internal resistance reduce charge storage efficiency and apparent capacitance. Consequently, reporting capacitance across a range of current densities or scan rates is essential for evaluating true rate capability and power performance. Normalization methodology further affects performance interpretation [34]. Capacitance may be reported on gravimetric (F/g), areal (F/cm^2^), or volumetric (F/cm^3^) bases, depending on the intended application. While gravimetric capacitance is commonly used in academic literature, areal and volumetric metrics are often more relevant for practical devices such as micro-supercapacitors and compact integrated systems. Similarly, energy and power densities can vary substantially depending on whether calculations are based solely on active material mass or the total packaged device mass [48]. Failure to clearly define normalization methods can lead to misleading comparisons and overestimation of practical performance.

### 3.2. Metal Oxides

Transition metal oxides have emerged as promising alternatives or additions to carbon materials due to their capability for fast surface redox reactions. Notably, ruthenium oxide (RuO_2_) and manganese dioxide (MnO_2_) have demonstrated remarkable pseudocapacitive properties. Hydrous RuO_2_ exhibits an exceptionally high theoretical specific capacitance in the range of 1400–2000 F/g, far surpassing typical carbon-based electrodes [3]. In practice, nanostructured RuO_2_ electrodes have achieved gravimetric capacitances around 700–1300 F/g. For example, studies on hydrous RuO_2_ nanotubes report capacitance around 1300 F/g with excellent charge and discharge characteristics [3]. RuO_2_ thin films can retain a high capacitance of around 1235 F/g at relatively fast potential sweep rates with high stability of >75% capacitance retention after thousands of cycles. Composite designs that layer RuO_2_ with conductive substrates like graphene or CNTs can exceed 1000 F/g while also improving durability, with one study showing around 92% capacitance retention after extensive cycling for a RuO_2_/graphene composite [3]. These reports explain why RuO_2_ is often considered a “gold standard” pseudocapacitive material, although its high cost and toxicity limit use to lab-scale demonstrations.

Manganese dioxide (MnO_2_) is another widely studied oxide due to its low cost and environmental friendliness relative to RuO_2_. MnO_2_ can provide specific capacitances on the order of a few hundred F/g and can be combined with carbon or other conductive supports to improve its typically poor electrical conductivity. MnO_2_-based electrodes suffer from structural changes and associated capacity fade during cycling, but their abundance makes them attractive for practical devices if these issues can be mitigated. Strategies such as using ultra-thin MnO_2_ on carbon scaffolds or combining multiple oxides are being explored to harness MnO_2_’s capacitance without sacrificing life.

An attraction of metal oxides is the relative ease of tuning their synthesis. For instance, a “one-pot hydrothermal” method where all reactions occur in a single sealed vessel under heated aqueous conditions can produce well-dispersed RuO_2_ nanostructures [3,11]. Furthermore, forming hybrid oxides or mixed-valence compounds can exploit synergistic effects. Blending RuO_2_ with MnO_2_ on a graphene substrate is one example; the graphene provides conductivity and a flexible support, RuO_2_ contributes high capacitance, and MnO_2_ adds cost-effectiveness, overcoming issues like particle agglomeration and mechanical rigidity [3].

Beyond RuO_2_ and MnO_2_, other transition metal oxides such as Fe_3_O_4_, V_2_O_5_, NiO, and Co_3_O_4_ have been investigated. Many show decent capacitance and unique electrochemical signatures, but none have matched RuO_2_’s combination of high capacitance and relatively easy kinetics. Often, less expensive oxides are employed in asymmetric configurations or as part of composites rather than as sole-electrode materials in symmetric cells. In summary, metal oxides can significantly increase the capacitance and thus energy storage of supercapacitors via faradaic processes. They typically offer higher specific capacitance than carbon alone, but lower cycle stability due to volumetric changes on redox cycling. Current research often focuses on composite approaches incorporating oxides with carbon or other matrices to balance these trade-offs.

### 3.3. Conducting Polymers and Composite Materials

Conducting polymers, such as polyaniline (PANI) and polypyrrole (PPy), have gained prominence as supercapacitor electrodes due to their high pseudocapacitance, intrinsic flexibility, light weight, and tunable electrical properties [49]. These organic materials undergo rapid redox doping and un-doping, contributing faradaic charge storage in a manner analogous to battery electrodes but typically with much faster kinetics. PANI, for example, has a theoretical capacitance up to 3400 F/g based on its multiple redox states [44]. In practice, PANI-based electrodes have demonstrated extremely high specific capacitances in the lab: a PANI-carbon quantum dot-CuO nanocomposite achieved 1070 F/g, and a PANI coating on activated carbon fiber/CNT yielded 5476 mF/cm^2,^ which translates to several hundred F/g [41]. Such high values illustrate the potential of conducting polymers. However, a well-known issue with polymers like PANI and PPy is cycling stability. Pure PANI tends to suffer from swelling or shrinkage and degradation of its polymer chains over repeated cycling, often resulting in significant capacitance loss after only a few thousand cycles.

To address this, conducting polymers are frequently used in composites with carbon materials. The carbon component, such as activated carbon, carbon nanofibers or CNTs, provides a stable scaffold and maintains conductivity, while the polymer provides the additional pseudocapacitance. Studies have shown that an optimal balance exists: a composite electrode with a small fraction of PANI, as low as 5–10% by mass, or even ~0.1% in some cases, can significantly boost capacitance while the carbon network preserves the polymer and prevents its mechanical disintegration [41]. The manufacturing processes for polymers are relatively tunable; chemical or electrochemical polymerization methods can yield various nano-morphologies (nanofibers, nanotubes, thin films) and can be combined with graphene or CNT additives that act as “stabilizers” to mitigate the polymer’s structural changes [50]. Overall, conducting polymers enable very high initial capacitance and can impart flexibility to the electrodes, but they are usually integrated as one component of a hybrid material to ensure longevity.

Beyond individual material classes, hybrid and composite materials have become a central focus for improving supercapacitors. By combining different types of materials, carbon for electric double-layer capacitance and conductivity, metal oxides or polymers for pseudocapacitance, researchers aim to overcome the limitations of each component [15]. For example, RuO_2_, carbon composites, as mentioned, pair the high capacitance of RuO_2_ with the stability and conductivity of carbon. Another example is integrating graphene with a lithium-ion battery anode material (like Li_4_Ti_5_O_12_) to create a lithium-ion capacitor, which behaves partly like a battery and partly like a supercapacitor. Such an “asymmetric” device can charge and discharge faster than a battery due to the EDLC-type graphene electrode while storing more energy than a pure supercapacitor, due to the battery-type electrode [15]. These hybrids effectively shift the device’s performance into the space between traditional capacitors and batteries.

Polymer/oxide composites are another sophisticated approach: for instance, PANI combined with MnO_2_ nanostructures or PPy combined with V_2_O_5_ can yield electrodes where both components contribute pseudocapacitance, and the composite architecture allows ionic pathways that maximize utilization of each component’s active sites [11]. The integrated structure maintains the advantageous characteristics of each component, rapid ion transport and mechanical resilience from the carbon or polymer matrix, and high charge storage from the faradaic material.

An important subclass of composites is metal sulfide-carbon nanocomposites. Metal sulfides such as NiS, CoS_2_, and CuS can have higher specific capacitances and energy densities comparable to oxides. However, many metal sulfides are unstable in air and suffer from poor cycle life. By combining metal sulfides with carbon, researchers have greatly enhanced their performance and stability. For example, a Ni_3_S_2_-reduced graphene oxide composite achieved 860 F/g at 5 mV/s [41]. A NiCuS/CNT composite delivered 1110 C/g (coulombic capacity) with an energy density of 52.5 Wh/kg at 750 W/kg [41]. Most impressively, multi-walled CNT/NiS_2_ nanocomposites demonstrated 2054 F/g at 2 A/g current [41]. These results show that carbon not only protects sulfides from oxidation but also increases surface area and creates synergistic effects that improve electron and ion transport, yielding exceptionally high capacitances.

Transition metal nitrides have also emerged as attractive electrode materials because of their metallic conductivity, chemical stability, and rapid charge-transfer behavior. Materials such as TiN, VN, MoN, and WN exhibit conductivity values approaching those of metals while simultaneously providing pseudocapacitive charge storage. Vanadium nitride (VN), in particular, has demonstrated capacitances exceeding 1000 F/g due to its fast reversible surface redox reactions and high electrical conductivity [51]. Additionally, nitrides often exhibit superior thermal and mechanical stability compared to conducting polymers or some oxides, making them promising for high-power and high-temperature applications. Nevertheless, surface oxidation and synthesis complexity remain significant challenges for widespread implementation. Emerging boron-based materials, particularly borophene and transition-metal borides, have attracted significant attention for supercapacitor applications due to their high electrical conductivity, tunable electronic structures, and strong electrochemical activity. Borophene offers exceptionally high theoretical quantum capacitance and rapid charge transport, while metal borides provide additional pseudocapacitive charge storage through fast multivalent redox reactions, enabling improved energy density and cycling stability [52]. Finally, it is worth mentioning the role of emerging 2D materials like graphene and MXenes, as discussed, as well as transition metal dichalcogenides within composites. These materials offer unique advantages, such as atomic thickness for short ion paths, high aspect ratio for percolation networks, but face challenges, including a tendency to restack, limited scalability, and sometimes lower conductivity. A common strategy is to incorporate such 2D materials into hybrid structures where, for instance, graphene provides conductivity for a metal oxide, or MXene provides pseudocapacitance in a carbon matrix [53]. As noted earlier, a single-layer graphene has an enormous surface area that can yield tremendous capacitance if fully utilized, theoretically a few hundred F/g per layer purely via double-layer charge [39], but practical devices require preventing layer restacking and maintaining electrolyte access.

In summary, composite and hybrid materials represent a pathway to engineer electrode materials that benefit from multiple charge storage mechanisms. By careful design, nanoscale mixing of components, scaffold-support relationships, protective coatings, and graded interfaces, researchers are crafting electrode materials that achieve higher energy densities while retaining high power and good lifespan. This material innovation is critical for next-generation supercapacitors that must meet diverse and demanding application requirements.

### 3.4. Emerging Systems

Emerging classes of materials, including two-dimensional metal–organic frameworks (2D MOFs), covalent organic frameworks (COFs), and high-entropy materials (HEMs), have attracted increasing attention for next-generation supercapacitor electrodes. These materials aim to overcome limitations associated with conventional carbons, metal oxides, and conducting polymers by simultaneously improving ion transport, electrical conductivity, and electrochemically active surface accessibility [54,55,56].

Two-dimensional metal–organic frameworks (2D MOFs) are particularly promising because their layered nanosheet morphology provides short ion diffusion pathways and abundant redox-active sites [56]. Unlike traditional bulk MOFs, which often suffer from poor conductivity and limited electrolyte accessibility, ultrathin 2D MOFs expose a greater fraction of active metal centers to the electrolyte and facilitate faster charge-transfer kinetics. Transition metals such as Ni, Co, Mn, and Fe are commonly incorporated into MOF structures to provide pseudocapacitive behavior through reversible redox reactions. Additionally, pyrolysis of MOFs can produce highly porous heteroatom-doped carbons or metal oxide-carbon hybrids with exceptional surface area and tailored pore distributions [57]. However, challenges remain regarding structural stability, conductivity, and scalable synthesis methods suitable for industrial production.

Covalent organic frameworks (COFs) represent another emerging class of crystalline porous materials composed entirely of light elements connected through strong covalent bonds. COFs possess highly ordered pore architectures, tunable functional groups, and low density, making them attractive for ion transport and charge storage applications. Unlike many MOFs, COFs generally exhibit superior chemical stability and lower density, which can benefit gravimetric energy storage performance [58]. Functionalization with redox-active groups such as quinones, amines, or sulfur-containing moieties can significantly enhance pseudocapacitive contributions. Furthermore, conductive COFs and COF-carbon composites have demonstrated improved electron transport while maintaining large accessible surface areas [59]. Despite these advantages, many COFs still suffer from intrinsically low electrical conductivity and complex synthesis requirements that limit commercial implementation.

High-entropy materials (HEMs), including high-entropy oxides, sulfides, and layered compounds, have recently emerged as highly promising electrode candidates due to their multi-element compositions and associated synergistic effects [60]. In these systems, five or more principal elements are incorporated into a single-phase structure, producing severe lattice distortion, enhanced defect concentrations, and multiple electrochemically active sites. These features can improve ionic diffusion, electrical conductivity, and structural stability during repeated charge–discharge cycling. High-entropy oxides based on combinations of Ni, Co, Mn, Fe, and Cu have demonstrated enhanced pseudocapacitive behavior and improved cycling retention compared to conventional single-metal oxides [61]. Additionally, the compositional flexibility of HEMs allows tuning of electrochemical properties for specific applications, including hybrid supercapacitors and flexible energy storage devices. However, the electrochemical mechanisms governing charge storage in high-entropy systems are not yet fully understood, and synthesis reproducibility remains an active area of research [62].

## 4. Mechanical and Functional Properties

In addition to electrochemical performance, supercapacitors must exhibit mechanical and environmental robustness, especially as their applications expand into wearable electronics, flexible sensors, electric vehicles, and other systems subject to physical stress, vibration, and shock. The mechanical and functional properties of the constituent materials largely determine a supercapacitor’s long-term reliability, performance consistency, and safety under real-world conditions. Key considerations include structural stability, flexibility, mechanical robustness under deformation, and thermal/chemical stability. This section examines these properties and how they influence supercapacitor design.

### 4.1. Structural Stability and Flexibility

Structural stability refers to the ability of electrode materials to maintain their morphology, conductivity, and pore structure throughout repeated charge/discharge cycling and handling. Because supercapacitors store energy primarily at the electrode surface, preserving a high surface area and an ion-accessible pore network is essential for maintaining capacitance and fast charge transport. Materials such as graphene, carbon nanotubes (CNT), and carbon nanofibers are commonly used not only for their electrochemical properties but also because they form robust, interconnected frameworks that can withstand mechanical strain [63]. For example, CNT or graphene-based electrodes can bend or fold with minimal cracking, thanks to the intrinsic flexibility of carbon allotropes and the strong interfacing between nanostructures. These flexible carbon frameworks provide efficient electron pathways and are resilient to stress, which is particularly important in emerging applications like foldable devices.

During prolonged cycling or compressive loading, porous high surface area electrodes can experience potential collapse. Supercapacitor performance relies on the interconnected pore network of porous electrodes, allowing for rapid ion transfer. However, repeated cycling and mechanical compression can cause pore walls to collapse, degrading performance by reducing effective surface area and decreasing capacitance [64]. In composite electrode systems, differences in thermal expansion of bonded materials can lead to separation and delamination. Similarly, differences in deformation tendency between the electrode and electrolyte can cause delamination in flexible systems, leading to failure [65].

The most common failure mechanism of supercapacitors is electrode cracking caused by repeated expansion and contraction during charge–discharge cycles. In pseudocapacitive materials and conducting polymers, ion insertion and redox reaction occur at the electrolyte electrode interface, generating localized mechanical stresses that can initiate microcracks [66]. After many cycles, the cracks can reach critical flaw size and propagate through the electrode, reducing electrical continuity. Polymers are especially susceptible to these reactions, causing swelling and shrinkage of polymer chains, leading to mechanical fatigue and fragmentation of the electrodes [67]. These complications decrease the active area, reducing capacitance and increasing internal resistance, which can lead to excess heating during cycles, accelerating the degradation process.

**Figure 3:**
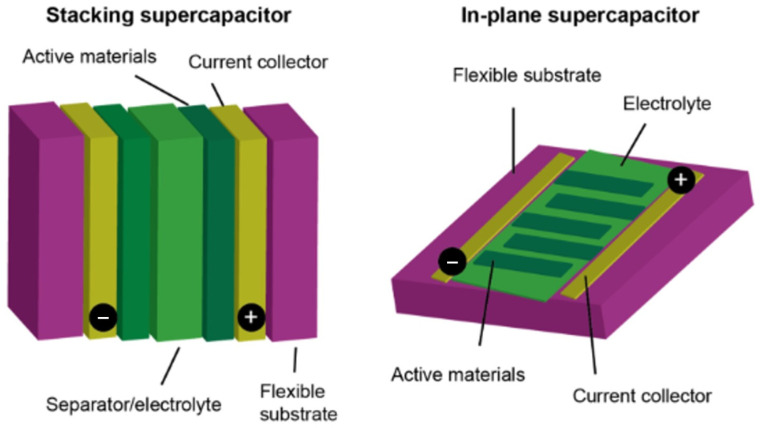
Supercapacitor design techniques, stacked and in-plane, reproduced from [71], Applied Sciences, MDPI 2022.

As supercapacitors move into flexible and wearable devices, electrode materials must tolerate frequent mechanical stress like bending, twisting, or stretching without performance loss. Engineers have developed novel architectures such as woven carbon textiles, aerogel-like foams, and layered MXene films that maintain electrical continuity during deformation [68]. Additionally, incorporating elastic polymer binders, polyurethane or styrene-butadiene rubber, can improve electrode cohesion and prevent delamination when the device is flexed [69]. The central design challenge is ensuring electrodes can withstand thousands of bending or stretching cycles while retaining their charge storage capability. Figure 3 shows a diagram of (left) a traditional capacitor architecture with stacked layers of active materials, and (right) shows an in-plane architecture allowing for deposition on a flexible substrate. Deposition can be done with a few methods, including direct printing. Studies have shown that certain flexible electrodes, such as CNT textiles, graphene foams, and carbon-cloth-based supercapacitors, can endure over 10,000 bending cycles with less than 10% loss in performance [70]. This level of fatigue resistance is crucial for wearable tech, where constant motion is expected.

Hybrid composite systems contribute to mechanical robustness. For instance, electrodes that combine carbon materials with pseudocapacitive components may face issues like brittleness if the pseudocapacitive material is not mechanically resilient. Researchers have developed strategies like nanostructured polymer reinforcements, gradient-layer coatings, and flexible current collectors to ensure that even when faradaic materials are present, the overall electrode can flex without damage [72]. Another frontier is textile-embedded energy storage: supercapacitor electrodes printed or coated directly onto fabrics. Such devices are appearing in health-monitoring garments, motion-capture suits, and smart textiles [73]. These applications impose additional requirements like resistance to mechanical abrasion, folding, and even washing. Consequently, durable and resilient electrode architectures often involving a combination of conductive fibers, protective coatings, and strain-accommodating structures are needed to preserve performance in these conditions.

### 4.2. Thermal and Chemical Stability

Thermal and chemical stability determine whether a supercapacitor can operate safely and effectively under varying environmental conditions. Many engineering systems experience significant temperature fluctuations, such as automotive under-hood environments, aerospace applications, or outdoor sensors in winter vs. summer. Commercial supercapacitors typically operate from about −40 °C up to +70 or +85 °C. Advanced designs, especially those using ionic liquid or solid-state electrolytes, can maintain stability at even higher temperatures (≥120 °C) [74].

Carbon-based materials are generally thermally stable and do not melt or decompose until very high temperatures in an inert atmosphere, and resist oxidative degradation reasonably well, especially if properly protected. In contrast, some pseudocapacitive materials can undergo phase changes or degrade at elevated temperatures. For example, MnO_2_ or RuO_2_ can undergo structural transformations or lose water if hydrous when heated. Repeated temperature cycling can introduce mechanical stresses due to expansion/contraction mismatch, leading to cracks unless mitigated by a composite structure [75]. Researchers often incorporate polymer matrices or nanoscale coatings to reinforce metal oxide electrodes, improving their thermal cycling stability.

The electrolyte is often the most temperature-sensitive component. Aqueous electrolytes freeze below 0 °C and can break the device, and they also boil/evaporate at high temperatures. Organic electrolytes like acetonitrile or propylene carbonate-based can operate up to 70 °C, but risk increased vapor pressure and degradation at higher temperatures. Ionic liquids have been touted for their wide liquid range; some remain liquid from −75 °C to +250 °C, and non-volatile nature, which dramatically improves high-temperature stability [76]. However, ionic liquids can be expensive and sometimes have higher viscosity, which can reduce power at low temperatures. Another approach is solid-state or gel polymer electrolytes, which incorporate the electrolyte into a solid or quasi-solid matrix such as PVA-H_3_PO_4_ gel, or a polymeric ionic conductor [77]. These gels do not leak, can be made flame-retardant, and can adhere to electrodes, which is beneficial for flexible devices. They also tend to have better thermal stability than liquids, preventing expansion or boiling.

Over time, electrode materials should resist chemical reactions with the electrolyte or environment. For example, activated carbon is quite inert, but at high potentials it can oxidize, especially in aqueous electrolytes, causing CO_2_ evolution. Metal oxides might dissolve or undergo valence changes if the potential window or pH is unfavorable. Ensuring a proper voltage window staying within the stable range of both electrodes and electrolyte is key to chemical stability. Additives in electrolytes or surface coatings like alumina on carbon or conductive polymers on oxides are sometimes used to create a protective interface that prevents unwanted reactions.

In summary, achieving thermal and chemical stability extends supercapacitor operating life and safety. It involves selecting materials that can endure the temperature extremes of the intended application and ensuring that both electrode and electrolyte remain inert to side reactions over time. This stability directly affects cycle life, which is one of supercapacitors’ primary advantages over batteries. A device that maintains performance over hundreds of thousands of cycles and wide temperature ranges is invaluable for reliable long-term operation in critical systems.

## 5. Fabrication and Processing Approaches

For supercapacitors to be viable in advanced energy systems, their fabrication processes must be scalable, cost-effective, and maintain high performance. Modern applications demand not only higher capacitance and energy/power densities, but also consistency, miniaturization, and integration into complex devices. This section reviews key fabrication and processing approaches: thin-film deposition techniques for precise, small-scale devices, printing and coating methods for large-scale manufacturing, and the challenges in processing that must be addressed for engineering applications.

### 5.1. Thin-Film Deposition

Thin-film fabrication is essential when precise control over electrode architecture is required, such as in micro-supercapacitors or when integrating energy storage into microelectronic systems. Ultra-thin electrodes maximize surface-to-volume ratios and minimize ion diffusion distances, which is ideal for power performance. Several deposition techniques are commonly used.

Physical Vapor Deposition (PVD), including evaporation, sputtering, and e-beam evaporation, can create dense films with nanometer-scale thickness control. PVD is often employed to deposit metal films or current collectors, but can also deposit carbon or metal oxide thin films. The resulting films have well-defined thickness and composition, useful for fundamental studies or microfabricated devices. However, PVD films may have low porosity, which can limit ion access, so often a subsequent step, like etching or templating, is needed to introduce pores, increasing surface area.

Chemical Vapor Deposition (CVD) is widely used for synthesizing carbon nanomaterials, notably graphene and CNT arrays, “carpets”. CVD can directly grow these materials on substrates, catalyst pads, in a conformal manner [78]. For instance, graphene can be CVD-grown as a continuous film, or CNTs can be grown vertically to form a high-surface-area electrode. CVD typically requires high temperatures and careful control of gas atmospheres, and often an additional transfer process if the growth substrate is not the final device substrate.

Atomic Layer Deposition (ALD) is a specialized CVD variant that allows layer-by-layer growth with angstrom-level precision. ALD is powerful for coating high-aspect-ratio structures uniformly. In supercapacitors, ALD can coat porous templates or deposit metal oxides like TiO_2_, Al_2_O_3_ as dielectrics, or even RuO_2_ with excellent conformity [19]. ALD-grown films are extremely uniform, which is advantageous for stability and for creating well-defined interfaces in hybrid devices. The drawback is that ALD has low throughput and is often limited to thin layers of a few tens of nanometers economically, so its use is targeted toward critical thin layers rather than bulk electrode fabrication.

Electrochemical Deposition (Electrodeposition) involves using an electric current to deposit material from a precursor solution onto a substrate. This is widely used for pseudocapacitive materials like MnO_2_ or conducting polymers because it can be done at room temperature and can directly yield porous nanostructured coatings. Electrodeposition can fill complex shapes and is relatively simple and scalable. It also avoids high-temperature steps, which helps when using flexible or polymer substrates [79]. A challenge is controlling the morphology and ensuring uniformity over large areas or multiple pieces for batch production.

Each thin-film approach has its niche. For micro-supercapacitors that might be integrated on a chip, thin-film methods like sputtering a RuO_2_ layer or CVD-growing a CNT forest provide the necessary precision and integration capability. When precision and control are essential, thin-film deposition is favored despite typically higher cost and lower throughput compared to printing techniques.

### 5.2. Printing, Coating, and Scalable Manufacturing

For larger-scale production of supercapacitor electrodes, especially for applications like vehicle modules, grid storage banks, or any scenario where many devices are needed, printing and coating techniques are attractive. These methods emphasize scalability and cost-effectiveness, even if some nanoscale precision is traded off.

**Figure 4:**
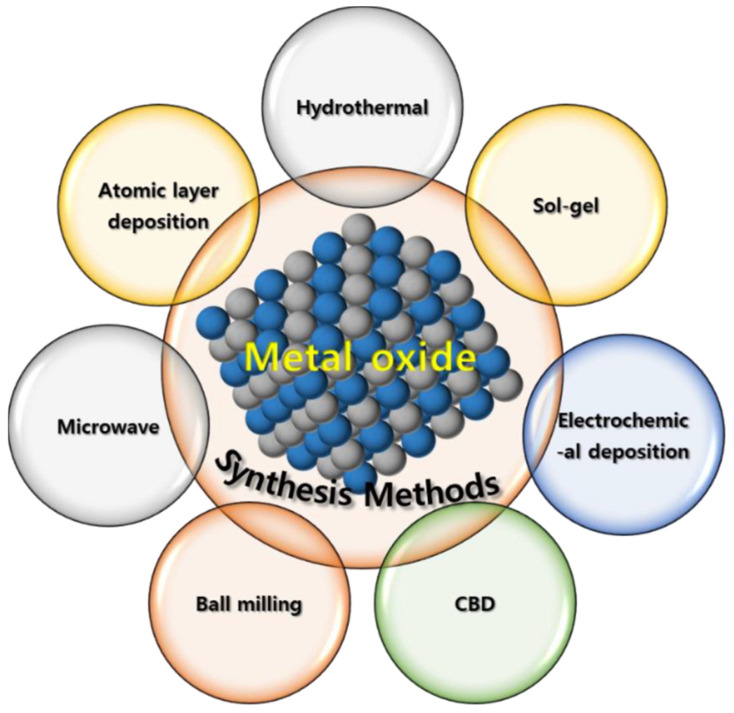
Metal oxide synthesis methods. Reproduced from [82], Nano Materials, MDPI 2022.

Screen Printing is a stencil-based approach where an ink containing activated carbon or MXene particles with a binder is pressed through a patterned screen onto a substrate. Screen printing can create precise electrode patterns on flexible substrates like plastics or textiles and is compatible with roll-to-roll processing. It is relatively low-cost and fast. Researchers have used screen printing to fabricate thin, flexible supercapacitor electrodes in predefined shapes, for example, interdigitated fingers for micro-supercapacitors [80]. Figure 4 shows a range of synthesis methods for metal oxides such as MnO_2_ and RuO_2_.

Inkjet Printing uses a computer-controlled printhead to deposit tiny droplets of ink in a chosen pattern. It offers digital design flexibility, no physical mask needed, and can produce high-resolution features on the order of tens of micrometers. In supercapacitors, conductive inks like graphene or silver nanowires for current collectors, and active material inks such as CNT or polymer solutions, can be printed layer by layer. Inkjet printing is attractive for prototyping and situations where the design may change frequently because it is maskless. However, it requires the ink to have certain fluid properties, such as viscosity, surface tension, and the feature sizes are limited by drop spread, typically around 50 µm or more [81].

Spray Coating/Aerosol Spraying technique atomizes an ink or suspension and sprays it onto a surface, often with a moving nozzle for uniform coverage. Spray coating can rapidly cover large areas and conformally coat complex surfaces. It tends to produce relatively uniform, thin films and can be used to deposit layers of activated carbon, CNTs, or MXenes onto substrates, even onto 3D shapes. An advantage is that spray deposition often yields porous, nano-structured films, which are beneficial for supercapacitor electrodes, and the process can be easily scaled, similar to paint spraying [80].

In research settings, vacuum filtration of nanomaterial dispersions like CNTs or MXenes through a membrane can produce a uniform film. The film, often called a “buckypaper” for CNTs or MXene film, can then be transferred onto a current collector. This method yields binder-free electrodes with densely packed nanomaterials and is good for achieving high gravimetric performance in small-scale cells, but it is not yet scalable for high-volume manufacturing.

Many of these methods are amenable to roll-to-roll processing, where electrodes are continuously formed on a flexible roll of substrate material and later assembled into devices. This greatly improves throughput, analogous to newspaper printing. A key challenge in scaling is ensuring consistent coating quality over large areas; small variations in ink formulation, substrate surface energy, or drying conditions can lead to performance variability. Quality control, including inline thickness measurement and uniformity monitoring, is thus critical.

### 5.3. Challenges in Processing for Engineering Applications

Despite the array of fabrication techniques available, several challenges must be overcome to integrate supercapacitors into engineering systems at scale. Large-area electrodes must have consistent thickness, loading, and composition. Minor inconsistencies can cause cell-to-cell performance variations or even internal imbalance in one cell (leading to local hot spots or accelerated degradation). For example, a roll-to-roll coated activated carbon electrode might vary by a few micrometers in thickness across the width; this needs to be minimized through precise coating controls. Techniques like ALD and high-quality CVD yield superb uniformity but are slow and costly; conversely, printing is fast but may introduce more variation. Scaling ALD or other precise methods to industrial throughput remains a challenge [83]. Thick electrodes or those deposited on flexible substrates may undergo stress due to swelling (ion insertion can cause expansion) or handling. During rapid charge/discharge, heating and electrostrain can cause subtle expansion/contraction. Over many cycles, this can crack a brittle film or cause delamination from the current collector. Engineering the interface and optimizing electrode thickness and porosity is needed to balance high active mass with mechanical integrity. As electrode thickness increases to achieve higher areal capacitance, ion transport can become rate-limiting, reducing power performance. Ensuring a proper pore size distribution (hierarchical porosity with micro-, meso-, and macropores) and not making films too dense is crucial. Some fabrication methods inherently produce dense films; PVD yields dense coatings with limited porosity, which might not be suitable for thick electrodes unless combined with post-processing. The way supercapacitors are packaged affects their performance and life. For instance, a flexible supercapacitor may be sealed in a polymer pouch. The packaging must prevent electrolyte drying and protect from the environment (moisture/oxygen ingress) over time. Processing steps must accommodate this. For example, after screen printing electrodes on foil, one must assemble and seal the device. This final assembly is as important as electrode fabrication. Poor seals can lead to self-discharge and short operational lifetimes. Techniques like ALD give high quality but low throughput; printing gives high throughput but requires careful optimization to approach the performance of lab-made small devices. Research is focused on speeding up precise methods, such as spatial ALD for faster deposition and improving printed electrode performance, for example, using wet-jet milling to prepare better graphene inks that produce higher conductivity films [83]. Table 4 summarizes some fabrication approaches, contrasting their capabilities and the challenges associated with each.

**Table 4:** Fabrication Methods and Considerations.

Approach	Thin-Film Technique (Mechanism)	Printing/Scalability	Engineering Challenges	Reference
PVD (e.g., thermal evaporation, sputtering)	Dense films, nm–µm scale; precise patterns possible	Wafer-scale deposition; clean vacuum process with high yield	Low porosity (limits ion access); not cost-effective for large areas	[84,85]
CVD (for graphene/CNTs)	Conformal growth of graphene or CNT “carpets” with edge-rich structure	Batch growth on substrates with catalyst, limited transfer steps	High temperature; need to transfer active material to device substrate; potential defects	[86]
Inkjet Printing	Deposits aerosolized droplets of carbon or conductive inks (~20–100 µm features)	Highly scalable via multi-head arrays; digitally programmable patterns	Feature size > 40 µm; ink formulation crucial for adhesion and conductivity	[81]
3D Printing (Direct Ink Writing)	Extrudes viscous ink to build layered 3D electrode structures	Enables custom architectures; relatively fast prototyping	Ink rheology must balance printability and stability; printed features often rough	[81]
Electrodeposition	Electrochemical formation of coatings (e.g., MnO_2_, conducting polymer) on complex shapes	Adapts to arbitrary substrate shapes; simple bath setups	Controlling morphology and avoiding impurities; thickness uniformity on large areas	[81]

## 6. Applications in Mechanical Engineering

### 6.1. Hybrid Electric Vehicles and Transportation

Hybrid and electric vehicles have stringent demands for both energy and power. During regenerative braking, for example, a vehicle experiences a short, intense burst of energy that must be captured quickly; similarly, during acceleration, a high power output is required briefly. Batteries alone struggle with such power spikes because they are designed for energy storage and have limitations in discharge current. Supercapacitors, with their high power density, are ideal to pair with batteries in these scenarios.

In a typical hybrid system, a battery-supercapacitor combination is used to harness each component’s strengths. During braking, the drive motor acts as a generator, and the surge of current is routed into a supercapacitor bank, which, due to its low internal resistance, can accept the high current without a large voltage rise [87]. A DC-DC converter often manages the power flow, buffering the supercapacitor’s voltage into the main DC bus. When the vehicle accelerates again, the supercapacitor supplies energy and power for the initial burst, providing the immediate thrust due to its superior power delivery, after which the battery takes over to supply the sustained energy once the peak demand subsides. This arrangement keeps the battery operating in a gentler regime, closer to a preferred state-of-charge and with lower current ramps, which reduces battery heating and prolongs its life. In effect, the supercapacitor handles the seconds-scale bursts, while the battery provides energy over longer periods (minutes to hours) [87].

**Figure 5:**
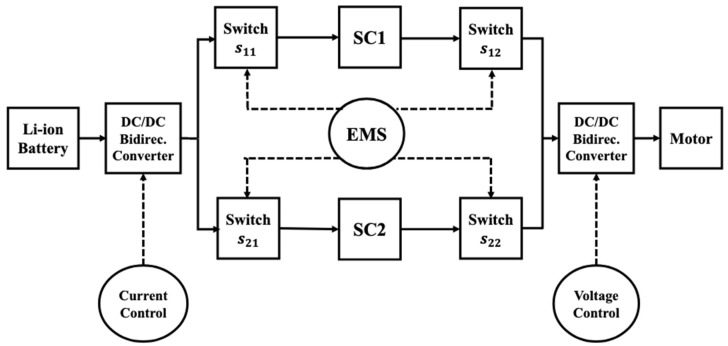
Schematic of a possible hybrid super capacitor/battery control system for EV. An energy management system (EMS) uses a set of switches to direct power to and from the super capacitors to minimize battery usage. Reproduced from [89], Sustainability, MDPI 2023.

The benefits of this hybrid approach include more efficient energy recovery, improved acceleration, and reduced stress on the battery, leading to longer battery lifetime and improved safety. A comparison can be made between the performance characteristics of a lead-acid battery and a supercapacitor, emphasizing that while the battery holds more energy, the supercapacitor can deliver/accept power much more rapidly [88]. Figure 5 shows a schematic of an electric vehicle (EV) power system using a Li-Ion battery in conjunction with supercapacitors and the necessary controllers to create a more efficient power delivery system. Relying solely on supercapacitors for propulsion is impractical due to their low energy density. However, when properly sized for peak power support, they dramatically enhance the overall system performance. In heavy-duty applications like buses or cranes, where frequent start-stop or lifting operations occur, supercapacitor modules are already used commercially to augment or replace batteries for power buffering.

By utilizing the complementary features of batteries and supercapacitors, hybrid energy storage systems in modern vehicles achieve better drivability, cooler battery operation, and improved overall efficiency during dynamic driving cycles. Supercapacitors in these systems are usually connected via power converters that manage their discharge–charge to the drivetrain, ensuring that the battery sees smoothed-out power demands. This reduces thermal stress and can allow batteries to be smaller or operated more conservatively. The result is a more efficient vehicle, extended component lifetimes, and potentially extended range because more braking energy is recuperated.

### 6.2. Portable and Wearable Electronics

Portable and wearable electronics demand thin, lightweight, and reliable energy storage that can be recharged frequently. Although batteries like Li-ion cells are commonly used, there is growing interest in micro-supercapacitors and flexible supercapacitors for these applications. The reason is that many wearable devices, fitness trackers, medical sensors, and electronic textiles do not actually need hours of energy storage. Instead, they need many quick charge/discharge cycles, fast charging, and a low risk of explosion or leakage. Supercapacitors align well with those needs.

Micro-supercapacitors are miniaturized supercapacitors, often with interdigitated electrodes on the scale of millimeters or less, that can be integrated on circuit boards or even directly on the chip [90]. They can supplement or replace electrolytic capacitors for power conditioning and can serve as tiny energy buffers for sensors. They maintain longer cycle life than batteries and can deliver high peak power from a small volume [91]. With the increasing demand for on-body and portable devices, there is a rapid development of energy storage devices that are ultra-lightweight and flexible [92]. There has been a recent development of biocompatible supercapacitors for in-body sensors and medical monitoring systems [93,94]. Supercapacitors are emerging as among the most promising flexible energy storage devices because they can endure millions of cycles while maintaining performance [95].

In wearable applications, supercapacitors often utilize materials spanning both EDLC carbons and pseudocapacitive phases to maximize performance. For instance, carbon nanotube or graphene-based fabrics can provide a flexible, conductive backbone, while incorporating a conducting polymer or a 2D material like MXene can raise the volumetric capacitance without sacrificing flexibility [70,95,96]. Hybrid electrodes such as graphene-MXene or CNT-PANI composites achieve high capacitance and maintain structural integrity under bending, a key requirement for wearables that experience constant motion.

Manufacturing techniques suited to wearables include screen-printing or inkjet-printing conductive inks onto textiles or polymer films to fabricate the electrode patterns [73]. Carbon inks with activated carbon or graphene and MXene inks have been successfully printed to make fully integrated flexible supercapacitors that can be attached to clothing or devices. These printed devices tend to be low-cost and can be made in arbitrary shapes, for example, printing a supercapacitor in the shape of a logo on clothing that also serves as an energy storage unit.

One limitation of supercapacitors in portable electronics is their self-discharge rate. A charged supercapacitor will lose charge over time on the order of days to weeks, faster than a well-made battery, which means if a wearable is left idle, the capacitor may drain. This can be mitigated by encapsulation and careful device design using separators that minimize leakage currents and encapsulating the device to prevent environmental factors from increasing self-discharge. For instance, a stretchy silicone encapsulant can be used to seal a flexible supercapacitor, keeping oxygen and moisture out, which can cause self-discharge via redox side reactions or electrolyte degradation and also serving as a strain-relief layer.

In summary, for portable and wearable electronics, supercapacitors offer the appealing combination of fast charging, long life, and robust operation, no need to replace after a few years like a battery, and less worry about deep discharge damage. They are finding use in devices like smart bands, wireless quick charge cases for earbuds, and even as energy harvesters combined with solar or kinetic energy converters on wearables, where they can store spurts of harvested energy and then supply sensors or transmitters.

### 6.3. Backup Power in Mechanical/Robotic Systems

**Figure 6:**
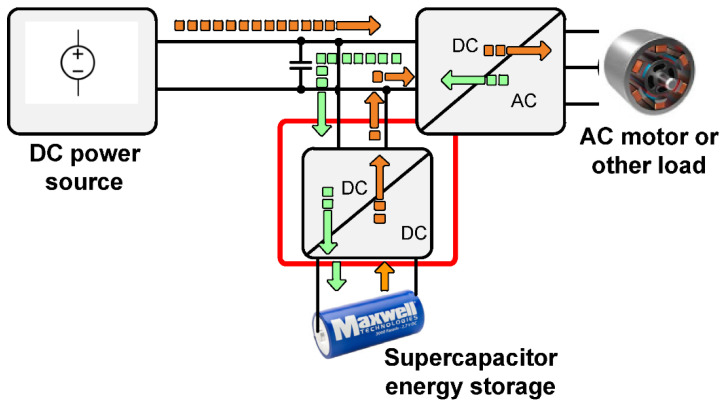
Diagram of a backup power system for an electric motor. Orange arrows indicate power delivery to the motor. Green arrows indicate power generated by the motor being directed to the supercapacitor for storage. Inside the red box is a DC/DC converter that steps the voltage up or down to charge the capacitor and drive the motor. Reproduced from [98] Energies, MDPI 2024.

In many mechanical and robotic systems, continuous power is provided by a primary source, usually mains electricity or a battery. However, critical failures often occur not from a total energy shortfall, but from a brief loss of power or a sudden surge in demand that upsets control systems or memory. Supercapacitors can be used as backup power or power smoothing devices in such contexts, due to their reliability in delivering short-term power. For example, consider an industrial robotic arm or a CNC machine; if there is a momentary power outage or voltage dip, it could cause the machine to lose reference or data and halt abruptly in a dangerous state. A supercapacitor module can provide the needed power for a few seconds or minutes to allow the system to either safely shut down or maintain operation until backup generators kick in. Figure 6 shows how a supercapacitor could be connected in line with power delivery to an electric motor, which could be powering the spindle or motion systems in the case of a CNC machine. Unlike batteries, supercapacitors do not mind being on constant standby and can be cycled millions of times, important if these events are frequent [97].

Emergency actuation is another scenario: certain mechanical systems require a quick action when power fails, for instance, a spring release, valve closure, or deploying a brake. Supercapacitors can discharge rapidly to drive actuators for these emergency operations. Think of a safety gate that must close immediately if power is lost, or a clutch that needs a burst of energy to engage a lock. These devices require instant high current, which supercapacitors can provide even if they have been trickle-charged and sitting charged for long periods.

In automated vehicles, cranes, and conveyor systems, large motors can draw huge currents during acceleration or lifting. To avoid straining the main power supply, which could cause brownouts or require oversizing the supply, supercapacitors are used to absorb or supply these peaks. They act as a buffer during a peak demand, where the motor draws from both the supply and the supercapacitor. Then, during light load or braking, excess energy charges the supercapacitor. This not only smooths the energy demand, improving efficiency and preventing voltage sag, but also protects components. In a crane, for example, when lowering a load, regenerative energy can charge supercapacitors that can be used when lifting, reducing the stress on the grid connection or diesel generator.

Large systems like wind turbines also use supercapacitors for backup and actuation. A wind turbine needs to have its blades pitched to a safe position if grid power is lost or in an emergency stop. Supercapacitors can provide the burst of power to the blade pitch motors to feather the blades and prevent damage. Similarly, they can help in the cold start of heavy machinery or turbines by providing a quick, high-energy source of power to get things moving.

Overall, the priorities for backup power applications are high reliability, immediate response, and predictable performance over many cycles or long calendar life. Supercapacitors fulfill these by being essentially maintenance-free once installed, capable of outputting power on demand with minimal delay, and they do not need cycling to stay healthy, unlike some battery chemistries. Their limited energy is not a drawback in these applications, since only short-duration power is required.

### 6.4. Smart Materials and Sensors Powered by Supercapacitors

The integration of supercapacitors with smart materials and sensors enables systems that can both sense, actuate, store, and deliver energy on demand. Often, smart sensors or actuators have intermittent high-power demands. For example, a sensor node might normally draw micro-watts but occasionally needs a burst of several milliwatts to transmit data, or a smart material might need a pulse of current to change state. Supercapacitors are ideal for providing these bursts. In magnetorheological (MR) dampers or electroactive actuators, the MR damper changes its viscosity and thus damping force when a magnetic field is applied, which requires a quick pulse of current through a coil. Similarly, shape memory alloy actuators need a surge of current to heat the alloy for it to change shape. These high-power pulses are short but crucial. A supercapacitor can be charged slowly from a battery or energy harvester and then be discharged quickly to supply the needed pulse, without stressing a small battery. This combination ensures that the device can perform the needed high-power action reliably every time, even if the primary source is low-power.

In remote or self-powered sensors, energy might be harvested slowly from the environment via solar cells or vibration harvesters. The harvested energy accumulates in a supercapacitor, then, every so often, the sensor wakes up, takes a measurement, and uses the stored energy to power a wireless transmission. After that, it goes back to charging mode. This energy harvesting and buffering model is very efficient and extends the lifetime of sensor nodes, potentially indefinitely, since no battery replacement is needed [99]. The supercapacitor can handle hundreds of thousands of these charge–discharge cycles with negligible degradation, which is perfect for maintenance-free sensor networks.

Another intriguing development is smart supercapacitors themselves, sometimes called structural supercapacitors or multi-functional capacitors if they also bear load or serve a secondary purpose. For example, electrochromic supercapacitors are devices that act both as an energy storage unit and a color-changing indicator. When charged or discharged, the material, often a conducting polymer or metal oxide, changes color, thereby visually displaying the state of charge [100]. This dual functionality can eliminate the need for external electronics or displays to monitor the device. In such devices, materials like polyaniline or tungsten oxide might serve as both the electrode and the electrochromic element, combined with a complementary carbon electrode to form a working supercapacitor [101]. These systems can be useful in smart windows or indicators that store energy and simultaneously provide information or dynamic functionality, like a window that tints when it stores solar energy.

More broadly, smart sensors with integrated power, sometimes referred to as “smart dust” or “autonomous sensors,” benefit enormously from supercapacitors. Without them, one might need a larger battery to handle the intermittent loads, leading to more weight, higher cost, and a finite lifetime. With a supercapacitor, the primary energy source can be something ultra-compact or energy harvesting only, and the device can be sealed for life with no battery swaps. In these applications, the material and design choices often revolve around combining the energy storage with the sensing-actuating function. Conductive polymers and metal oxides again play a role, as they can sometimes serve as the functional material while also being part of the capacitor. The challenge is balancing the two roles: for example, a material that is optimized for changing color might not store as much charge, so designers either accept a trade-off or design the device cleverly.

In summary, supercapacitors enable smart materials and sensors to operate more effectively by providing quick energy bursts and by potentially adding multifunctionality. This synergy opens up design possibilities for self-sustaining, fast-response smart systems in the Internet of Things, structural health monitoring, and active control systems.

## 7. Challenges and Future Directions

The development of supercapacitor technologies has been rapid in recent years, and supercapacitors show great promise as a cornerstone in modern energy systems. However, several challenges and open questions remain before supercapacitors can realize their full potential across all applications. Many of these challenges are not just scientific but also practical and economic. This section discusses key issues such as the fundamental energy–power trade-off, cost and scalability hurdles, environmental considerations, and novel integration strategies, as well as likely future directions of research.

### 7.1. Trade-Off Between Energy and Power Density

One of the central challenges in supercapacitor development is the trade-off between energy density and power density. Supercapacitors are well known for their excellent power delivery capabilities and long life spans, tolerating extensive charge cycles with minimal degradation, but they still trail far behind batteries in terms of energy density [101]. This disparity means that for applications requiring sustained energy output over long durations, supercapacitors cannot yet replace batteries. As discussed earlier, improving energy density (Wh/kg) often comes at the cost of power or cycle life. For instance, using thicker electrodes or pseudocapacitive materials can increase energy storage, but may reduce the achievable power or the number of cycles due to diffusion limits or material changes.

**Figure 7:**
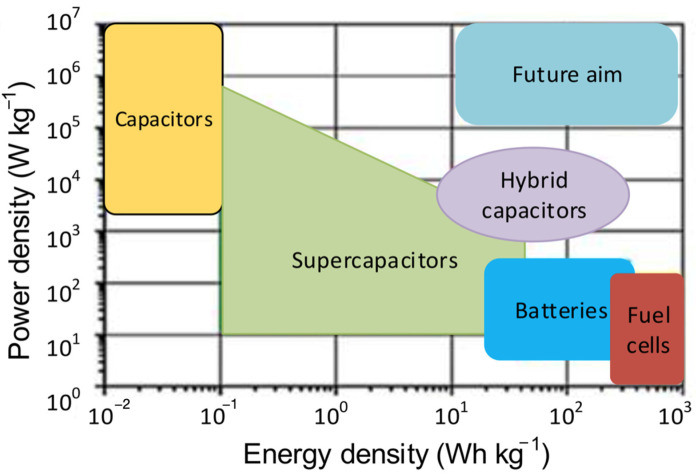
Power vs. Energy density of notable energy storage technologies. Reproduced from [102] energies, MDPI 2022.

Figure 7 illustrates this balance by showing a Ragone plot of various energy storage technologies [102]. Supercapacitors occupy the high-power, low-energy corner, whereas batteries occupy the high-energy, lower-power region. Bridging this gap is a primary focus of current supercapacitor research. Techniques such as developing new electrode materials and hybrid devices aim to push supercapacitors rightward on the Ragone plot without losing much on the vertical axis. To date, some lab-scale devices have achieved energy densities on par with lead-acid batteries around 20–30 Wh/kg while still providing >10× the power density of those batteries, but commercial supercapacitors are still typically in the 5–10 Wh/kg range [20,26].

It is also important to note that supercapacitors do not experience capacity fade when left unused, as many batteries do. Batteries can self-degrade over calendar time even if not cycled. This is an advantage that cannot be captured in a Ragone plot but matters in real usage. For most applications, the single biggest limitation of supercapacitors is still total energy storage. Ongoing research is trying to address this through material science and device engineering.

An aspect of the energy vs. power challenge is the operating voltage window. Energy density scales with the square of cell voltage (E ∝ ½CV^2^). Traditional EDLC supercapacitors have a max cell voltage around 2.7–3.0 V, limited by organic electrolyte decomposition. If one could increase this voltage, energy would increase significantly. This is why researchers look at ionic liquid electrolytes with some chemistries stable up to 3.5–4 V or solid-state electrolytes. Asymmetric configurations that use battery-type electrodes can operate at different potential ranges. The development of stable high-voltage supercapacitors is a parallel approach to improving energy density.

In summary, while supercapacitors have carved out a niche for short-burst, high-power applications, achieving the versatility to handle longer-duration energy supply without losing their inherent advantages remains a future goal. It is likely that rather than completely eliminating this trade-off, engineers will continue to tailor hybrid solutions combining supercapacitors with batteries or enhancing them with battery-like characteristics depending on the application’s needs.

### 7.2. Cost and Scalability

For any energy technology to achieve widespread adoption, it must be economically viable at scale. Currently, one of the significant challenges for supercapacitors is the material and manufacturing cost relative to the energy provided. On a cost-per-energy-stored basis ($/kWh), supercapacitors are generally more expensive than batteries. Analysis has shown that while supercapacitors might be competitive or even cheaper in terms of cost per power, they lag in cost per energy [5]. This limits their appeal for applications where energy is the dominant requirement, like grid storage for many hours, despite being attractive for power-centric roles.

The most significant material cost component in typical supercapacitors is often the activated carbon of the electrodes [5]. While carbon itself is not expensive, producing high-quality activated carbon with the necessary properties and consistency has a cost. Additionally, some advanced electrode materials like carbon nanotubes, graphene, or metal oxides are costly to produce or employ on a large scale. As the technology scales up, it is expected that economies of scale and improvements in production will bring down these costs. For instance, if biomass-derived carbons such as those from coconut shells or other agricultural waste can be processed in bulk to yield reliable supercapacitor-grade material, the cost could drop substantially.

Scalability in manufacturing also involves adapting processes for mass production. Current supercapacitor manufacturing for devices from a few Farads up to 5000 F cells uses techniques not unlike battery manufacturing, mixing powders, coating electrodes, and assembling in a case. These processes are well-established and can be scaled, but any introduction of new materials or designs requires re-validation at scale. Ensuring consistent performance for millions of cells is non-trivial; small variations in material, like a slightly different pore distribution in carbon, can affect performance, so quality control and process refinement are crucial.

Another factor is that supercapacitors currently fill a somewhat niche market. They often play a complementary role to batteries in applications like regenerative braking and short-term UPS, rather than a primary role. The market size, while growing, is smaller than that of batteries. Increased demand and production volume, for example, if future hybrid vehicles widely adopt supercapacitors, or if grid frequency regulation starts using them in bulk, will naturally bring costs down through scale.

From a technology strategy perspective, efforts are underway, such as the “Storage Innovations 2030” assessments, to identify key areas to reduce cost. These include developing cheaper electrode materials like those from biomass or more abundant metals for pseudocapacitors, simplifying manufacturing by printing instead of sophisticated fabrication, and increasing the energy content so that fewer supercapacitor cells are needed for a given application, thus distributing fixed costs like packaging across more energy.

In summary, while supercapacitors are technically impressive, their broader adoption will depend on driving down costs. This will likely happen by a combination of material innovation and scaling up production. Until then, supercapacitors will be used where their unique performance justifies the cost. As cost barriers lower, one can expect them to see more mainstream energy storage markets, possibly even in consumer electronics or grid storage, where currently batteries dominate.

### 7.3. Environmental Sustainability

Energy technologies today must also be evaluated on their environmental footprint. In this regard, supercapacitors have both positive aspects and some challenges compared to batteries. On the positive side, supercapacitors are often more environmentally benign in terms of materials. They largely consist of carbon, aluminum for current collectors or casings, and relatively small amounts of other materials. In contrast, many batteries rely on heavy metals or scarce elements like lead, cobalt, nickel, and lithium, whose mining and refining have significant environmental and social impact, such as habitat destruction, water contamination, and sometimes human rights issues in mining. Using activated carbon derived from renewable biomass or waste, such as coconut shell carbon or carbons from other agricultural byproducts, underscores the potential for a more sustainable supply chain for supercapacitors [103]. Moreover, the electrolyte in many supercapacitors, typically acetonitrile or propylene carbonate with a salt, can be hazardous if spilled, but it is not as problematic as lead in lead-acid batteries or flammable electrolytes in Li-ion cells. It should be noted that acetonitrile is flammable too, but newer capacitors often use propylene carbonate, which is safer.

Another environmental advantage of supercapacitors is their long life, which means fewer replacements and thus less waste over time. A supercapacitor that lasts 1,000,000 cycles might effectively last the lifetime of the equipment it is in, whereas a battery might be changed several times. This reduces the waste and resource consumption associated with manufacturing multiple battery replacements. Additionally, supercapacitors have a strong safety profile; they are far less prone to catastrophic failure like fires or explosions than high-energy batteries. Battery fires not only pose immediate danger but also release toxic fumes and require special handling for disposal. Supercapacitors, by virtue of storing energy physically and lacking reactive chemistries that can run away, are much safer in that regard [104].

However, supercapacitors are not without environmental impacts. The manufacturing processes, like high-temperature activation of carbon, or the use of KOH/acid in activation, solvent use in electrode processing, consume energy and can produce chemical waste. The carbon footprint of production can be significant unless renewable energy or cleaner processes are used. Moreover, while supercapacitor materials are more benign, recycling them is still challenging. Recycling a supercapacitor involves recovering aluminum, possibly recycling the organic electrolyte, and dealing with carbon powder. There is ongoing research into recycling processes.

One futuristic advantage could be the use of fully biodegradable or carbon-neutral components. For example, researchers are exploring using biodegradable electrolytes like ionic liquids derived from biomaterials or polymers that can serve as an electrolyte and then naturally decompose. Also, if the electrodes are made from biomass carbon and the device is properly recycled, the net environmental impact can be lowered significantly [21].

From a sustainability perspective, if the electricity used to charge supercapacitors comes from renewable sources, then an application like grid support by supercapacitors is very clean. They do not emit anything during use. Of course, one must also consider that widespread adoption of supercapacitors would mean manufacturing millions of them, which scales up the consumption of whatever materials they use. It appears that the material base of mostly carbon and aluminum can handle scale-up with relatively low environmental risk compared to scaling up cobalt or lithium mining for batteries.

Overall, supercapacitors are generally more environmentally friendly than many battery technologies currently in use. They use more abundant materials, have a longer lifespan, less frequent replacement, and pose fewer risks of pollution and accidents. The challenges lie in making their production greener, using renewable energy in manufacturing, avoiding hazardous chemicals in processing, and establishing recycling streams for end-of-life devices. Continued research in using sustainable materials like biomass carbons, aqueous electrolytes, and biodegradable components will further enhance the environmental profile of supercapacitors.

### 7.4. Integration into Structural and Flexible Systems

One of the exciting directions for supercapacitors is their integration into the fabric of devices and structures, essentially blurring the line between an energy storage device and the system it powers. This concept is often referred to as structural energy storage or, specifically, structural supercapacitors when applied to capacitors.

The idea behind structural supercapacitors is to use components that serve dual functions: they carry mechanical load and store electrical energy [105]. For example, in an electric vehicle, instead of having a separate heavy battery pack plus a chassis, one could have the car’s frame or body panels store energy. This would save weight and volume while potentially improving overall efficiency. Supercapacitors are particularly well-suited for this because of their stability and safety. Embedding a battery inside structural components raises more concerns about thermal runaway or complicated battery management; a supercapacitor can be more straightforward.

A typical structural supercapacitor might consist of carbon-fiber composites where the carbon fiber acts as one electrode and also bears mechanical load as it normally would in a composite, a separator/ionic conductor as the matrix for example, a solid polymer electrolyte that also binds the fibers, and another carbon fiber layer as the counter-electrode [106]. The result can be a carbon-fiber-reinforced plastic part that also stores charge. Carbon fibers are an attractive electrode for this because they have good electrical conductivity and decent surface area, especially if their surface is activated or coated with something like CNTs or graphene nanoplatelets to increase area and pseudocapacitance [107]. Additionally, certain polymer resins used in composites can be modified to conduct ions by adding ionic liquid or gel electrolyte characteristics, making them serve as both structural resin and electrolyte.

Recent research has shown some promising results; structural supercapacitor prototypes have been built that can power small devices while bearing load. For instance, a drone frame made with a structural supercapacitor could supply part of the power needed for its flight, reducing the battery weight it needs to carry. Challenges remain in optimizing both mechanical properties and electrical properties. The thickness of structural components might be a lot larger than ideal electrode spacing, etc., and ensuring good contact and low resistance across large, structural electrodes is non-trivial.

Advanced manufacturing techniques are key to making structural supercapacitors practical. Resin Transfer Molding (RTM) and its variant vacuum-assisted RTM (VARTM) are common composite manufacturing processes where resin is infused into fiber preforms [108]. Infusing a functional resin that acts as an electrolyte into a carbon fiber layup that is arranged properly to form electrodes could produce an integrated structure without much change to standard composite manufacturing. Similarly, additive manufacturing can play a role: direct ink writing can print conductive patterns or even entire electrode frameworks within a structural part’s geometry [109]. As 3D printing of functional materials advances, printing a complex shape that is both a load-bearing part and has an internal supercapacitor network could become common.

The balance between mechanical and electrochemical performance is a central design consideration. Typically, making a composite good at storing energy involves adding porosity or thinner separators for more capacitance, which might weaken it mechanically. Conversely, bulking it up for strength might reduce capacitance. The future direction of this research is to find sweet spots or multifunctional materials that inherently satisfy both needs. For example, carbon fiber fabrics coated with nanostructured electrode materials could provide the necessary surface area without entirely sacrificing fiber cross-section for mechanical duties. In flexible electronics as well, integrating supercapacitors “invisibly” into devices, such as a supercapacitor integrated into the casing of a phone, or into the fabric of a wearable, will be a trend. It requires collaboration between mechanical design and electrical design, far beyond simply housing a component; the component becomes part of the design structure itself.

In summary, integrating supercapacitors into structural and flexible systems represents a paradigm shift in how we think about energy storage, from a distinct component to a distributed function within materials. This could lead to significant weight and space savings and enable power in systems that cannot accommodate traditional batteries or capacitors. Achieving this will require continued innovation in materials like multifunctional carbon fiber composites, solid electrolytes, and fabrication methods, but the potential impact is substantial for industries ranging from aerospace to consumer gadgets to automotive.

## 8. Conclusions

Supercapacitors represent a rapidly advancing class of energy storage devices that effectively bridge the performance gap between traditional capacitors and batteries. Their ability to deliver high power density, withstand extensive charge/discharge cycling, and operate with high efficiency makes them essential in modern engineering applications ranging from regenerative braking systems to wearable electronics and autonomous robotic systems. While EDLCs, pseudocapacitors, and hybrid systems each offer unique advantages, ongoing research continues to push the boundaries of their energy density, mechanical durability, and scalability.

Material selection remains at the core of supercapacitor development. Carbon-based materials offer lightweight structures, high conductivity, and long cycle life; metal oxides and conducting polymers can significantly increase capacitance through fast redox reactions; and hybrid/composite materials combine these benefits, yielding electrodes that are both mechanically stable and electrochemically active. At the same time, advancements in fabrication thin-film deposition for micro-scale devices, printing/coating methods for large-scale production, and novel manufacturing for structural integration are making it more feasible and cost-effective to deploy supercapacitors widely.

Several challenges, however, still need to be addressed. Improving energy density without sacrificing power or cycle life remains a central focus, whether through new materials like 2D MXenes or redox-active frameworks, better electrolytes with wider voltage windows, or device design. Reducing manufacturing costs and ensuring consistent quality at scale are crucial for wider commercial adoption. Environmental sustainability is another key consideration; developing supercapacitors with greener materials and processes will enhance their appeal as an eco-friendly alternative to batteries. Furthermore, integrating supercapacitors into structural and flexible systems will require continued innovation in multifunctional materials to ensure they can bear loads or conform to shapes while storing energy.

Despite these challenges, the future of supercapacitor technology is bright. The combination of technical improvements and broadening application demands suggests that supercapacitors will play an increasingly important role in the evolving landscape of energy storage. For mechanical engineers, in particular, supercapacitors offer a toolbox of solutions for making systems more efficient, responsive, and durable. As devices and machines become smaller, smarter, and more energy-conscious, the need for fast, robust, and adaptable energy storage grows. Supercapacitors are well-positioned to meet industry needs. Continued interdisciplinary research and engineering innovation will undoubtedly shape the next generation of supercapacitors, solidifying their place in future energy storage portfolios.

## Figures and Tables

**Table 1:** General comparison between energy storage mediums.

Characteristic	Battery	Supercapacitor	Reference
Recharge Cycle Lifetime	<10^3^ cycles	>10^6^ cycles	[1]
Self-discharge Rate (per month)	~5%	~30%	[1]
Typical Cell Voltage	3.7–4.2 V	0–2.7 V	[3]
Energy Density (Wh/kg)	High (20–150)	Low (0.8–10)	[4]
Power Density (W/kg)	Low (50–300)	High (500–4000)	[1]
Fastest Charge Time	Minutes to hours	Seconds to minutes	[5]
Fastest Discharge Time	0.3–3 h	Under a few minutes	[1]
Charging Circuit Complexity	Complex	Simple	[1]

**Table 2:** Comparison of EDLCs, Pseudocapacitors, and Hybrid Supercapacitors (Data compiled from [1,2,3,4]).

Feature	EDLC (Electric Double-Layer Capacitor)	Pseudocapacitors	Hybrid Supercapacitor	Reference
Storage Mechanism	Electrostatic ion adsorption (double-layer)	Fast surface redox reactions	Combination of electrostatic + redox	[1]
Typical Electrode Materials	Activated carbon, CNTs, graphene	Transition metal oxides, conducting polymers	Carbon + metal oxide or carbon + polymer composites	[14]
Reversibility	Extremely high (physical charge storage)	Moderate to high (material dependent)	Moderate to high (material dependent)	[14]
Energy Density	Low to moderate	Moderate to high	Intermediate to high	[7]
Power Density	Very high	High	High	[7]
Cycle Life	Excellent (hundreds of thousands of cycles)	Lower due to structural changes	Intermediate	[28]
Primary Limitation	Limited energy density	Mechanical/chemical instability	More complex design and fabrication	[28]

**Table 3:** Key Supercapacitor Electrode Materials: Properties and Applications.

Material Type	Specific Capacitance (F/g)	Energy Density (Wh/kg)	Power Density (W/kg)	Cycle Life	Typical Test Conditions	Typical Applications	Reference
Carbon-based (EDLC) (e.g., Activated Carbon, Graphene, CNTs)	~100–300 (up to ∼500+ with advanced carbons)	Low (∼2–10) Limited by double-layer mechanism	Very High (∼1000–5000)	>100,000 cycles (excellent stability)	6 M KOH or 1 M H_2_SO_4_ aqueous electrolyte; 0–1 V; 0.5–5 A/g	High-power burst applications, regenerative braking, UPS systems, memory backup, wearable sensors (with flexible carbons)	[41]
Metal Oxides (Pseudocapacitive) (e.g., MnO_2_, RuO_2_)	∼200–1000 (RuO_2_ up to 1400+)	Moderate (∼5–20) Higher via faradaic reactions	High (∼500–2000)	10,000–100,000 cycles (limited by structural changes)	1 M Na_2_SO_4_ or KOH; 0–0.8 V; 1–10 A/g	Research prototypes requiring higher energy than EDLCs; memory backup; often combined with carbon in commercial devices to boost performance	[3]
Conducting Polymers (Pseudocapacitive) (e.g., PANI, PPy)	High (theoretical > 2000; practical 500–1000)	Moderate (∼5–15)	Moderate to High (∼500–1500)	Few thousand cycles (improving with composites)	Acidic aqueous electrolyte; 0–0.8 V; 0.5–5 A/g	Flexible/stretchable electronics, wearable devices, sensors; usually as coatings or composites due to poor standalone stability	[42]
Composite/Hybrid Materials (e.g., Carbon + Oxide, Carbon + Polymer, Asymmetric Capacitors)	Intermediate to High (∼300–1500, depending on constituents)	Higher (∼10–50) Bridging toward battery range	High (>1000, often 2000+)	Intermediate (≥ 50,000 cycles typical)	Organic or aqueous electrolyte; asymmetric cells; 1–20 A/g	Electric/hybrid vehicles (energy & power needs), grid energy buffering, heavy machinery (peaks shaving), advanced devices that demand both high energy & power	[44]

## Data Availability

No new data were created or analyzed in this study. Data sharing is not applicable to this article.
